# Synthesis and structure−activity relationship of 8-substituted protoberberine derivatives as a novel class of antitubercular agents

**DOI:** 10.1186/1752-153X-7-117

**Published:** 2013-07-10

**Authors:** Ying-Hong Li, Hai-Gen Fu, Feng Su, Li-Mei Gao, Sheng Tang, Chong-Wen Bi, Yu-Huan Li, Yan-Xiang Wang, Dan-Qing Song

**Affiliations:** 1 Institute of Medicinal Biotechnology, Chinese Academy of Medical Sciences and Peking Union Medical College, Beijing 100050, China; 2 College of Chemical Engineering, Qingdao University of Science and Technology, Qingdao 266042, China

**Keywords:** 8-Substituted-protoberberine, Antitubercular, Structure−activity relationship, Drug−resistance

## Abstract

**Background:**

The emergence of multi-drug resistant tuberculosis (MDR-TB) has heightened the need for new chemical classes and innovative strategies to tackle TB infections. It is urgent to discover new classes of molecules without cross-resistance with currently used antimycobacterial drugs.

**Results:**

Eighteen new 8-substituted protoberberine derivatives were synthesized and evaluated for their anti-mycobacterial activities against *Mycobacterium tuberculosis* (*M. tuberculosis*) strain H_37_Rv. Among them, compound **7g** was the most effective antitubercular agent with minimum inhibitory concentration (MIC) of 0.5 μg/mL. Moreover, it also afforded a potent antitubercular effect against clinically isolated MDR strains of *M. tuberculosis* with MICs ranging from 0.25 to 1.0 μg/mL, suggesting a novel mode of action.

**Conclusions:**

The structure−activity relationship (SAR) analysis revealed that introduction of a substituent at the 8-position in pseudoprotoberberine, especially an *n*-decyl, could significantly enhance the anti-TB activity. We consider 8-*n*-decylberberines to be a novel family of anti-tubercular agents with an advantage of inhibiting MDR strains of *M. tuberculosis*.

## Background

Currently, one third of the world’s population is infected with *Mycobacterium tuberculosis* (*M. tuberculosis*) [1]. It is anticipated that there will be about 8.9−9.9 million new and relapse tuberculosis (TB) cases this year, more than in any other year in history [2,3]. The limited effectiveness and long-term treatment lead to poor patient compliance, which often causes multi-drug resistant (MDR) and extensively-drug-resistant (XDR). The emergence of new cases, the increased incidence of MDR strains of *M. tuberculosis*, the adverse effects of first-line anti-TB drugs isoniazid (INH) and rifampin (RIF) [4,5], and the increased incidence of TB associated with HIV infections [6-8] have led to renewed research interest in discovering novel anti-TB drugs. Especially, the emergence of MDR-TB and of the virtually untreatable MDR-TB has heightened the need for new chemical class and innovative strategies to tackle TB infections. However, truly novel antitubercular drugs other than repurposed drugs have not been developed since the 1970s [9,10]. Though new anti-TB drug Bedaquiline was just approved by FDA last year [11], there is still an urgent need to discover new classes of molecules without cross-resistance with currently used antimycobacterial drugs.

**Figure 1:**
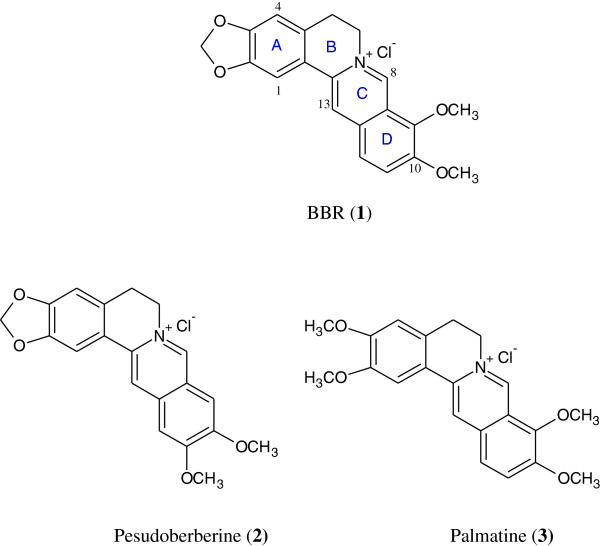
Chemical structures of compounds 1, 2 and 3.

**Figure 2:**
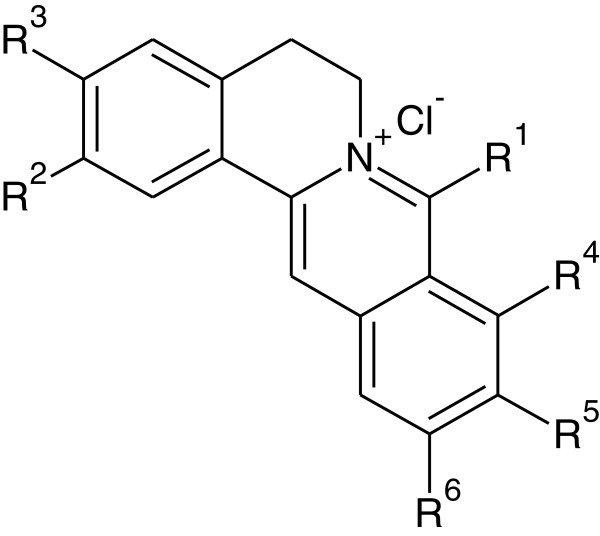
General structural formula of 8-substituted BBR derivatives.

We have identified 13-substituted protoberberine derivatives to be a novel family of anti-TB agents [12] with poor solubility. The primary structure−activity relationship (SAR) indicated that the berberine ring (BBR, **1**, Figure 1) might be beneficial for keeping good antitubercular activity. In our ongoing efforts to discovering new anti-TB agents, we turned our SAR analysis on the substituents at the 8-position of BBR derivatives, by which nitrogen ion at the 7-position might be blocked by the 8-substituents with bigger volume, thereby enhancing the solubility of this kind of compounds. Based on this strategy, several 8-substituted protoberberine derivatives (**6a**–**e**) were designed, semi-synthesized and evaluated for their antimycobacterial activity against *M. tuberculosis* strain H_37_Rv. Furthermore, by replacing **1** with pseudoberberine (**2**, Figure 1) or palmatine (**3**, Figure 1) core, two natural products extracted from Chinese herb Huanglian, by which a group of new 8-substituted pseudoberberine (**7a**–**h**) or palmatine (**8a**–**e**) derivatives was generated for testing. Herein, eighteen 8-substituted protoberberine derivatives (Figure 2) were designed and synthesized, and their anti-mycobacterial effects were evaluated afterwards.

## Results and discussion

### Chemistry

**Scheme 1:**
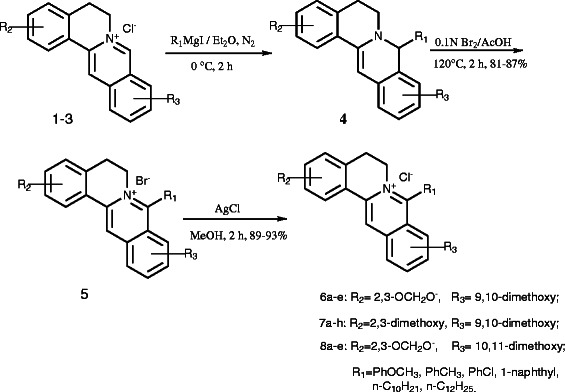
The synthetic route of the target compounds.

Eighteen target compounds were synthesized with commercially available **1**, **3** or **2** (synthesized in our laboratory) [13,14] as the starting material as described in Scheme 1. The Grignard reagents were first prepared with Mg turnings and the corresponding alkyl and aryl iodide in absolute ether under N_2_ protection. The key intermediates dihydroberberine, dihydropseudoberberine or dihydropalmatine (**4**) were obtained via nucleophilic substitution of newly synthesized Grignard reagents with **1**, **2** or **3** under N_2_ protection, respectively [15,16]. Then, the intermediate **4** was oxidized using bromine as a oxidizing agent in HOAc at refluxing temperature to yield the 8-substituted berberine bromate **5**, which was converted into the corresponding chloride **6**–**8** with AgCl in MeOH at room temperature. Finally, the desired products in series **6**, **7** and **8** were purified by flash column chromatography using methanol/dichloromethane as the gradient eluent with overall yields of 72%–81%.

### Biological activity and SAR analysis of 8-substituted protoberberine derivatives for anti-mycobacterial activity

Our SAR strategy was first focused on the modifications of the substituents at the 8-position in BBR. According to our previous SAR results, several lipophilic groups including *p*-methoxyphenyl (**6a**), *m*-methoxyphenyl (**6b**), *p*-methylphenyl (**6c**), 1-naphthyl (**6d**) and *n*-decyl (**6e**) were introduced into the 8-position aiming to improve the cLogP value (Table 1), thereby enhancing the anti-mycobacterial activity. Among these analogues, compound **6e** possessing an *n*-decyl afforded the hightest antibacterial activity with a MIC of 2.0 μg/mL against *M. tuberculosis*. The results supported that the increased cLogP value might be helpful for enhancing the anti-TB activity of this kind of compounds.

**Table 1:** **SAR of 8-substituted protoberberine analogous against *M. tuberculosis *** **strain H**_**37**_**Rv**

**Compd**^**a**^	**R**^**1**^	**R**^**2**^	**R**^**3**^	**R**^**4**^	**R**^**5**^	**R**^**6**^	**MIC**^**b**^	**cLogP**^**c**^
**1**							> 128	−0.77
**2**							128	−1.08
**3**							> 128	−0.77
**6a**	PhOCH_3_-*p*	OCH_2_O		OCH_3_	OCH_3_	H	32	0.35
**6b**	PhOCH_3_-*m*	OCH_2_O		OCH_3_	OCH_3_	H	16	0.35
**6c**	PhCH_3_-*p*	OCH_2_O		OCH_3_	OCH_3_	H	16	0.93
**6d**	1-naphthyl	OCH_2_O		OCH_3_	OCH_3_	H	8	1.60
**6e**	*n*-C_10_H_21_	OCH_2_O		OCH_3_	OCH_3_	H	2	3.29
**7a**	PhOCH_3_-*p*	OCH_2_O		H	OCH_3_	OCH_3_	4	0.35
**7b**	PhOCH_3_-*m*	OCH_2_O		H	OCH_3_	OCH_3_	2	0.35
**7c**	PhCH_3_-*p*	OCH_2_O		H	OCH_3_	OCH_3_	2	0.93
**7d**	PhCl-*p*	OCH_2_O		H	OCH_3_	OCH_3_	1	1.14
**7e**	PhCl-*m*	OCH_2_O		H	OCH_3_	OCH_3_	2	1.14
**7f**	1-naphthyl	OCH_2_O		H	OCH_3_	OCH_3_	2	1.60
**7g**	*n*-C_10_H_21_	OCH_2_O		H	OCH_3_	OCH_3_	0.5	3.29
**7h**	*n*-C_12_H_25_	OCH_2_O		H	OCH_3_	OCH_3_	1	4.35
**8a**	PhOCH_3_-*p*	OCH_3_	OCH_3_	OCH_3_	OCH_3_	H	16	0.04
**8b**	PhOCH_3_-*m*	OCH_3_	OCH_3_	OCH_3_	OCH_3_	H	16	0.04
**8c**	PhCH_3_-*p*	OCH_3_	OCH_3_	OCH_3_	OCH_3_	H	16	0.62
**8d**	1-naphthyl	OCH_3_	OCH_3_	OCH_3_	OCH_3_	H	4	1.29
**8e**	*n*-C_10_H_21_	OCH_3_	OCH_3_	OCH_3_	OCH_3_	H	1	2.98
RIF							0.0625	3.90
INH							0.0625	−1.12

^a^: General structural formular is shown in Figure 2.

^b^: MIC: minimum inhibitory concentration.

^c^: Chemoffice Ultra 11.0 (Cambridge office).

RIF: rifampin; INH: isoniazid.

In order to further explore the influence of the BBR core, a variety of lipophilic substituents were attached to the 8-position of **2**, by which the 8 new 8-substituted pseudoberberine classes (**7a**−**h**) were generated for testing. The results showed (Table 1) that the majority of them (**7b**−**h**) exhibited potential anti-mycobacterial activities with MICs ranging from 0.5 μg/mL to 2.0 μg/mL. It seems that the 10,11-dimethoxy on the ring D would be beneficial for their binding affinity to the target molecular. Compound **7g** bearing an *n*-decyl afforded the best anti-TB activity with a MIC of 0.5 μg/mL. Similarly, the lipophilic side-chains were also introduced to the same position in **3**, and then 5 new 8-substituted palmatine analogues (**8a**−**e**) were made. As expected, compound **8e** with an *n*-decyl at position 8 had the most potent activity with an MIC of 1.0 μg/mL. It was deduced that an *n*-decyl at the 8-position would improve the anti-mycobacterial activity as regarding to this kind of compounds. The pesudoberberine ring might be beneficial for the antimycobacterial activity, and thus the representative compounds in **7** series were chosen for further investigation.

### Anti-resistance TB effect of 7g and 7f

As compound **7g** bearing an *n***-**alkyl at position 8 possessed an excellent activity against drug-susceptible *M. tuberculosis* strain H_37_Rv, it was selected to test the anti-TB activity against MDR strains. In this experiment, *M. tuberculosis* strains 87, 192, 262 and 266 isolated from the patient infected with tuberculosis in China, were resistant to both RIF and INH. RIF and INH showed a decreased activity against the drug-resistant stains partially or completely with MIC ranges between 2 and > 32 μg/mL (Table 2), while compound **7g** afforded a potential effect against MDR strains with comparable MIC ranges of 0.25−1 μg/mL. In addition, compound **7f** possessing an aromatic moiety at the 8-position in **7** series afforded a moderate cLogP value (cLogP = 1.60) and then was chosen to evaluate for the drug-resistant strains as well. As described in Table 2, **7f** showed an equivalent potency against the drug-susceptible strain H_37_Rv and multidrug-resistance isolates of *M. tuberculosis* strains 257, 373, 559 and 164 with a MIC range between 2 and 4 μg/mL as well. The results indicated that **7g** and **7f** were effective for drug-susceptible *M. tuberculosis* as well as MDR strains isolated from TB patients in China, suggesting a mode of action different from currently used anti-TB drugs.

**Table 2:** ***In vitro *** **antitubercular activities of compounds 7f and 7g against MDR strains of *M. tuberculosis***^**a **^**(MIC: μg/mL)**

**Compd**	**257/87**	**373/192**	**559/262**	**164/266**	**H**_**37**_**Rv**
**7f**^**b**^	2	4	2	2	2
**7g**^**c**^	0.5	0.25	0.5	1	0.5
RIF	> 32	> 32	> 32	> 32	0.0625
INH	2	4	2	2	0.0625

^a^: MDR strains were isolated from patients with tuberculosis in China.

^b^: MIC values against MDR strains 257, 373, 559 and 164.

^c^: MIC values against MDR strains 87, 192, 262 and 266.

### Cytotoxicity of 7g and 7f in Vero and MRC-5 cells

Both of compounds **7g** and **7f** were further tested their cytotoxicity in African green monkey kidney (Vero) and human lung fibroblast (MRC-5) cells with MTT assay. Cytotoxicity activity was expressed with CC_50_ value, and the selectivity index (SI), as an important therapeutic indication, was calculated as the ratio of CC_50_ to MIC value. Anti-TB effect of compounds **7g** and **7f** was evaluated by combining their MIC with SI values. As described in Table 3, compound **7g** showed a moderate SI value of 10.3 and 17.6 in Vero and MRC-5 cells, respectively.

**Table 3:** **Cytotoxicity**^**a **^**activities of compounds 7f and 7g in Vero and MRC-5 cells**

**Compd**	**Vero**		**MRC-5**	
	CC_50_ (ug/ml)	SI^b^	CC_50_ (ug/ml)	SI
**7f**	14.72 ± 2.02	7.37	10.18 ± 2.78	5.09
**7g**	5.15 ± 1.29	10.3	8.78 ± 0.48	17.6

^a^: Cytotoxic concentration required to inhibit Vero or MRC-5 cell growth by 50%.

^b:^ Selectivity index (SI) value equaled to CC_50_/MIC.

### Experimental

#### Instruments

Melting point (m.p.) was uncorrected and recorded on a Mettler Toledo MP90 melting point apparatus. ^1^H NMR and ^13^C NMR spectra were recorded on Varian 400 MHz spectrometer. Chemical shift was reported relative to internal tetramethylsilane (δ 0.00 ppm) or (CD_3_)_2_SO (δ 2.50 ppm) for ^1^H and (CD_3_)_2_SO (δ 39.5 ppm) for ^13^C. ESI high-resolution mass spectra (HRMS) were recorded on an Autospec UItima-TOF mass spectrometer (Micromass UK Ltd, Manchester, UK). Flash chromatography was performed on CombiflashRf 200 (Teledyne, Nebraska, USA), particle size 0.038 mm.

#### General procedure to obtain final 8-substituted protoberberine derivatives

Grignard reagents were prepared via Magnesium turnings (3.8 g) with the corresponding alkyl and aryl iodides (0.13 mol) in absolute ether (100 mL) at 0°C. The synthesized Grignard reagents were added to the suspension of dry **1**, **2** or **3** (0.03 mol) in absolute ether (100 mL) dropwise under N_2_ protection at 0°C [15,16]. After refluxing for 2 h, saturated NH_4_Cl solution (200 mL) was added to quench the reaction. The aqueous phase was extracted with ethyl acetate (3 × 100 mL) and the combined organic layers were washed with saturated brine (100 mL) and dried (Na_2_SO_4_). The mixture was concentrated in vacuo to give **4**. Then, bromine solution (1N/ HOAc, 30 mL) was added to oxidize compound **4** in HOAc at refluxing temperature to generate the 8-substituted BBR derivatives bromate **5**, which was treated with excessive AgCl in MeOH at room temperature thus converted into the corresponding chloride form. Finally, the desired products in series **6**, **7** and **8** were purified by flash column chromatography using methanol/ dichloromethane as the gradient eluent with overall yields of 72%–81%.

#### 8-(p-Methoxy)phenylprotoberberine choride (6a)

Yield: 78%; Brown solid; mp 220–221°C; ^1^H NMR: δ 9.04 (s, 1H), 8.23 (d, *J* = 9.2 Hz, 1H), 8.11 (d, *J* = 9.2 Hz, 1H), 7.84 (s,1H), 7.56 (d, *J* = 8.8 Hz, 2H), 7.22 (d, *J* = 8.8 Hz, 2H), 7.04 (s,1H), 6.18 (s,2H), 4.26 (t, *J* = 6.8 Hz, 2H), 4.00 (s, 3H), 3.98 (s, 3H), 3.20 (s, 3H), 3.01(t, *J* = 6.8 Hz, 2H); ^13^C NMR: δ 172.40, 160.65, 156.96, 153.08, 150.26, 148.18, 145.55, 138.47, 133.66, 131.30, 129.73, 126.90, 126.30, 124.72, 122.84, 121.88, 121.81, 114.41, 110.01, 108.36, 106.31, 102.56, 61.09, 57.44, 55.90, 52.26, 26.67; HRMS-ESI: *m/z* calcd 442.16490 C_27_H_24_NO_5_Cl [M − Cl]^+^, found 442.16476.

#### 8-(m-Methoxy)phenylprotoberberine choride (6b)

Yield: 79%; Orange solid; mp 206–207°C; ^1^H NMR: δ 9.07 (s, 1H), 8.24 (d, *J* = 9.2 Hz, 1H), 8.11 (d, *J* = 9.2 Hz, 1H), 7.85 (s, 1H), 7.58 (t, *J* = 8.0 Hz, 1H), 7.20-7.26 (m, 3H), 7.04 (s, 1H), 6.18 (s, 2H), 4.27 (t, *J* = 6.4 Hz, 2H), 4.00 (s, 3H), 3.81 (s, 3H), 3.26 (s, 3H), 3.02 (t, *J* = 6.4 Hz, 2H); ^13^C NMR: δ 172.49, 159.61, 156.25, 153.02, 150.31, 148.20, 145.40, 138.37, 136.15, 133.72, 131.28, 130.41, 126.41, 124.65, 122.44, 121.76, 120.09, 115.85, 113.70, 108.39, 106.29, 102.58, 61.15, 57.45, 55.87, 52.35, 26.66; HRMS-ESI: *m/z* calcd 442.16490 C_27_H_24_NO_5_Cl [M − Cl]^+^, found 442.16466.

#### 8-(p-Methyl)phenylprotoberberine choride (6c)

Yield: 76%; Orange solid; mp 158–160°C (decomp); ^1^H NMR: δ 9.05 (s, 1H), 8.23 (d, *J* = 9.2 Hz, 1H), 8.12 (d, *J* = 9.2 Hz, 1H), 7.85 (s, 1H), 7.52 (d, *J* = 8.4 Hz, 2H), 7.47 (d, *J* = 8.4 Hz, 2H) ,7.03 (s, 1H), 6.18 (s, 2H), 4.23 (t, *J* = 6.4 Hz, 2H), 3.99 (s, 3H), 3.17 (s, 3H), 3.01 (t, *J* = 6.4 Hz, 2H), 2.47 (s, 3H); ^13^C NMR: δ 172.47, 156.95, 153.04, 150.28, 148.19, 145.48, 139.84, 138.45, 133.68, 132.14, 131.27, 129.59, 129.50, 128.03, 127.94, 126.38, 124.68, 122.63, 121.83, 108.37, 106.30, 102.57, 61.04, 57.46, 52.32, 26.63, 21.60; HRMS-ESI: *m/z* calcd 426.16998 C_27_H_24_NO_4_Cl [M − Cl]^+^, found 462.16986.

#### 8-(1-Naphthyl)protoberberine choride (6d)

Yield: 72%; Brown solid; mp 175–176°C; ^1^H NMR: δ 8.82 (s, 1H), 8.15−8.32 (m, 3H), 7.95−8.07 (m, 1H), 7.64−8.00 (m, 5H), 7.45−7.55 (m, 1H), 7.03 (s, 1H), 6.21 (s, 2H), 4.11−4.30 (m, 2H), 4.10 (s, 3H), 3.98 (s, 3H), 2.90−3.05 (m, 2H); ^13^C NMR: δ 172.49, 156.87, 152.61, 150.73, 148.43, 145.38, 139.24, 136.72, 132.16, 131.87, 130.76, 130.08, 129.20, 128.26, 127.56, 126.01, 125.12, 124.39, 123.76, 121.65, 120.95, 108.39, 106.68, 106.29, 106.09, 102.69, 60.99, 57.91, 52.56, 26.81; HRMS-ESI: *m/z* calcd 462.16998 C_30_H_24_NO_4_Cl [M − Cl]^+^, found 462.16996.

#### 8-(n-Decyl)protoberberine chloride (6e)

Yield: 80%; Brown solid; mp 185–186°C; ^1^H NMR: δ 8.81 (s, 1H), 8.20 (d, *J* = 9.2 Hz, 1H), 8.02 (d, *J* = 9.2 Hz, 1H), 7.74 (s, 1H), 7.12 (s, 1H), 6.17 (s, 2H), 4.80 (t, *J* = 6.4 Hz, 2H), 4.03 (s, 3H), 4.05 (s, 3H), 3.16 (t, *J* = 6.4 Hz, 2H), 1.78 (t, *J* = 6.4 Hz, 2H), 1.27−1.58 (m, 16H), 0.87 (t, *J* = 6.4 Hz, 3H); ^13^C NMR: δ 172.49, 161.58, 152.99, 150.09, 148.11, 146.08, 138.29, 133.10, 131.28, 125.66, 125.20, 121.92, 120.71, 108.19, 106.26, 102.48, 62.05, 57.52, 50.10, 32.77, 31.78, 29.83, 29.46, 29.22, 29.19, 28.40, 27.13, 22.58, 21.54, 14.45; HRMS-ESI: *m/z* calcd 476.27954 C_30_H_38_NO_4_Cl [M − Cl]^+^, found 476.27971.

#### 8-(p-Methoxy)phenylpseudoprotoberberine choride (7a)

Yield: 79%; Bright orange solid; mp 197–198°C; ^1^H NMR: δ 8.87 (s, 1H), 7.79 (s, 1H), 7.68 (s, 1H), 7.66 (d, *J* = 8.4 Hz, 2H), 7.33 (d, *J* = 8.4 Hz, 2H), 7.07 (s, 1H), 6.70 (s,1H), 6.19 (s, 2H), 4.34 (t, *J* = 6.4 Hz, 2H), 4.09 (s, 3H), 3.92 (s, 3H), 3.71 (s, 3H), 3.04 (t, *J* = 6.4 Hz, 2H); ^13^C NMR: δ 161.47, 157.40, 155.03, 152.41, 150.32, 148.17, 139.55, 136.77, 131.66, 131.56, 131.39, 123.39, 122.89, 121.94, 119.24, 116.98, 115.36, 108.50, 106.94, 106.22, 106.13, 102.55, 57.23, 56.27, 55.99, 52.12, 26.82; HRMS-ESI: *m/z* calcd 442.16490 C_27_H_24_NO_5_Cl [M − Cl]^+^, found 442.16488.

#### 8-(m-Methoxy)phenylpseudoprotoberberine choride (7b)

Yield: 77%; Yellow solid; mp 224–225°C; ^1^H NMR: δ 8.90 (s, 1H), 7.80 (s, 1H), 7.67−7.71 (m, 2H), 7.30−7.35 (m, 2H), 7.26 (d, *J* = 6.4 Hz, 1H), 7.08 (s, 1H), 6.66 (s, 1H), 6.19 (s, 2H), 4.33 (t, *J* = 6.4 Hz, 2H), 4.09 (s, 3H), 3.85 (s, 3H), 3.70 (s, 3H), 3.06 (t, *J* = 6.4 Hz, 2H); ^13^C NMR: δ 160.14, 157.53, 154.44, 152.48, 150.37, 148.20, 139.48, 136.92, 132.26, 131.74, 131.34, 122.93, 121.79, 121.62, 119.33, 117.31, 115.15, 108.54, 106.72, 106.20, 106.08, 102.57, 57.25, 56.27, 55.99, 52.27, 26.78; HRMS-ESI: *m/z* calcd 442.16490 C_27_H_24_NO_5_Cl [M − Cl]^+^, found 442.16475.

#### 8-(p-Methyl)phenylpseudoprotoberberine choride (7c)

Yield: 78%; Yellow solid; mp 212–213°C; ^1^H NMR: δ 8.89 (s, 1H), 7.80 (s, 1H), 7.69 (s, 1H), 7.61 (d, *J* = 8.4 Hz, 2H), 7.58 (d, *J* = 8.4 Hz, 2H), 7.07 (s, 1H), 6.65 (s, 1H), 6.19 (s, 2H), 4.31 (t, *J* = 6.4 Hz, 2H), 4.09 (s, 3H), 3.69 (s, 3H), 3.05 (t, *J* = 6.4 Hz, 2H), 2.54 (s, 3H); ^13^C NMR: δ 157.44, 155.01, 152.43, 150.34, 146.65, 148.18, 141.42, 139.55, 136.85, 131.35, 130.50, 129.66, 128.20, 123.13, 121.87, 119.30, 108.51, 106.80, 106.21, 106.14, 102.56, 57.24, 56.25, 52.20, 26.78, 21.64, 21.52; HRMS-ESI: *m/z* calcd 426.16998 C_27_H_24_NO_4_Cl [M − Cl]^+^, found 426.16986.

#### 8-(p-Chloro)phenylpseudoprotoberberine chloride (7d)

Yield: 75%; Bright orange solid; mp 183–184°C; ^1^H NMR: δ 8.91 (s, 1H), 7.94 (d, *J* = 8.0 Hz, 2H), 7.87 (d, *J* = 8.0 Hz, 2H), 7.75−7.80 (m, 1H) 7.69 (s, 1H), 7.08 (s,1H), 6.60 (s, 1H), 6.19 (s, 2H), 4.30 (t, *J* = 6.4 Hz, 2H), 4.09 (s, 3H), 3.72 (s, 3H), 3.06 (t, *J* = 6.4 Hz, 2H); ^13^C NMR: δ 157.55, 153.59, 152.59, 150.41, 148.22, 139.64, 136.97, 134.87, 132.64, 131.83, 130.97, 130.20, 129.89, 126.50, 123.05, 121.76, 119.55, 108.56, 106.50, 106.22, 105.06, 102.60, 57.28, 56.39, 52.38, 26.76; HRMS-ESI: *m/z* calcd 446.11536 C_26_H_21_NO_4_Cl_2_ [M − Cl]^+^, found 446.11483.

#### 8-(m-Chloro)phenylpseudoprotoberberine chloride (7e)

Yield: 76%; Bright yellow solid; mp 179–180°C; ^1^H NMR: δ 8.93 (s, 1H), 7.79−7.90 (m, 5H), 7.71 (s, 1H), 7.08 (s, 1H), 6.57 (s, 1H), 6.20 (s, 2H), 4.32 (t, *J* = 6.4 Hz, 2H), 4.09 (s, 3H), 3.71 (s, 3H), 3.07 (t, *J* = 6.4 Hz, 2H); ^13^C NMR: δ 157.58, 152.94, 152.63, 150.44, 148.23, 139.58, 137.05, 134.69, 132.96, 131.98, 131.74, 131.35, 129.59, 128.64, 123.00, 121.69, 119.63, 108.57, 106.40, 106.23, 106.14, 102.61, 57.31, 56.36, 52.46, 26.76; HRMS-ESI: *m/z* calcd 446.11536 C_26_H_21_NO_4_Cl_2_ [M − Cl]^+^, found 446.11481.

#### 8-(1-Naphthyl)pseudoprotoberberine choride (7f)

Yield: 72%; Yellow-brown solid; mp 198–200°C (decomp); ^1^H NMR: δ 9.14 (s, 1H), 8.38 (d, *J* = 7.8 Hz, 1H), 8.22 (d, *J* = 7.8 Hz, 1H), 7.84−7.92 (m, 4H), 7.67−7.71 (m, 1H), 7.51−7.55 (m, 1H), 7.40 (d, *J* = 8.2 Hz, 1H), 7.05 (s, 1H), 6.34 (s, 1H), 6.20 (s, 2H), 4.20−4.32 (m, 2H), 4.10 (s, 3H), 3.43 (s, 3H), 2.93−3.05 (m, 2H); ^13^C NMR: δ 157.14, 152.66, 152.16, 149.89, 147.72, 139.79, 136.83, 133.09, 131.62, 130.90, 130.17, 129.01, 128.92, 128.36, 127.50, 127.35, 125.86, 124.42, 123.19, 121.54, 119.55, 108.07, 105.90, 105.80, 105.65, 102.12, 56.85, 55.65, 51.55, 26.40; HRMS-ESI: *m/z* calcd 462.17053 C_30_H_24_NO_4_Cl [M − Cl]^+^, found 462.17072 (See Additional file 1).

#### 8-(n-Decyl)pseudoprotoberberine chloride (7g)

Yield: 78%; Yellow-brown solid; mp 173–174°C; ^1^H NMR: δ 8.67 (s, 1H), 7.68 (s, 2H), 7.11 (s, 1H), 6.37 (s, 1H), 6.17 (s, 2H), 4.77 (t, *J* = 6.4 Hz, 2H), 4.06 (s, 6H), 3.67 (t, *J* = 6.4 Hz, 2H,), 3.15 (t, *J* = 6.4 Hz, 2H), 1.24−1.16 (m, 16H), 0.85 (t, *J* = 4.0Hz, 3H); ^13^C NMR: δ 158.04, 157.19, 152.67, 150.12, 148.07, 139.33, 136.11, 131.31, 122.12, 122.02, 118.54, 108.31, 106.60, 106.21, 106.15, 102.49, 57.11, 56.94, 49.80, 31.77, 29.54, 29.45, 29.43, 29.26, 29.18 (2), 28.22, 27.17, 22.57, 14.44; HRMS-ESI: *m/z* calcd 476.28008 C_30_H_38_NO_4_Cl [M − Cl]^+^, found 476.27990 (See Additional file 1).

#### 8-(n-Dodecyl)pseudoprotoberberine chloride (7h)

Yield: 80%; Bright yellow solid; mp 161–163°C (decomp); ^1^H NMR: δ 8.65 (s, 1H), 7.66 (s, 1H), 7.59 (s, 1H), 7.09 (s, 1H), 6.36 (s, 1H), 6.16 (s, 2H), 4.75(t, *J* = 6.4 Hz, 2H), 4.05 (s, 6H), 3.65 (t, *J* = 6.4 Hz, 2H,), 3.14 (t, *J* = 6.4 Hz, 2H), 1.20−1.74 (m, 20H), 0.84 (t, *J* = 4.0Hz, 3H); ^13^C NMR: δ 158.12, 157.18, 152.67, 150.12, 148.07, 139.30, 136.09, 131.28, 122.09, 121.99, 118.50, 108.30, 106.57, 106.17, 106.12, 102.50, 57.10, 56.93, 49.79, 31.78, 29.53, 29.49, 29.42, 29.32, 29.25, 29.17, 29.14, 28.84, 28.21, 27.16, 22.58, 14.44; HRMS-ESI: *m/z* calcd 504.31084 C_32_H_42_NO_4_Cl [M − Cl]^+^, found 504.31047.

#### 8-(p-Methoxy)phenylprotopalmatine choride (8a)

Yield: 81%; Brown solid; mp 168–169°C; ^1^H NMR: δ 9.13 (s, 1H), 8.24 (d, *J* = 9.2 Hz, 1H), 8.14 (d, *J* = 9.2 Hz, 1H), 7.67 (s, 1H), 7.58 (d, *J* = 8.2 Hz, 2H), 7.23 (d, *J* =8.2 Hz, 2H), 7.04 (s, 1H), 4.29 (t, *J* = 6.4 Hz, 2H), 4.00 (s, 3H), 3.96 (s, 3H), 3.86 (s, 3H), 3.82 (s, 3H), 3.24 (s, 3H), 3.05 (t, *J* = 6.4 Hz, 2H); ^13^C NMR: δ 172.49, 160.74, 156.93, 152.77, 151.98, 149.36, 145.94, 139.98, 138.68, 133.77, 131.53, 129.72, 126.78, 124.63, 122.78, 121.49, 120.32, 113.38, 114.51, 111.29, 109.69, 61.32, 57.96, 56.81, 56.42, 55.87, 55.92, 26.37; HRMS-ESI: *m/z* calcd 458.19620 C_28_H_28_NO_5_Cl [M − Cl]^+^, found 458.19613.

#### 8-(m-Methoxy)phenylprotopalmatine choride (8b)

Yield: 78%; Red-brown solid; mp 170–171°C; ^1^H NMR: δ 9.15 (s, 1H), 8.25 (d, *J* = 9.2 Hz, 1H), 8.14 (d, *J* = 9.2 Hz, 1H), 7.76 (s, 1H), 7.56−7.60 (m, 1H), 7.20−7.26 (m, 3H), 7.05 (s, 1H), 4.29 (t, *J* = 6.4 Hz, 2H), 4.00 (s, 3H), 3.96 (s, 3H), 3.86 (s, 3H), 3.82 (s, 3H), 3.24 (s, 3H), 3.05 (t, *J* = 6.4 Hz, 2H); ^13^C NMR: δ 165.70, 159.62, 156.25, 152.87, 152.04, 149.24, 145.37, 138.59, 136.24, 133.82, 130.42, 129.30, 126.42, 124.54, 122.36, 121.52, 120.10, 115.78, 113.73, 111.21, 109.66, 61.13, 57.42, 56.73, 56.34, 55.87, 52.49, 26.35; HRMS-ESI: *m/z* calcd 458.19619 C_28_H_28_NO_5_Cl [M − Cl]^+^, found 458.19615.

#### 8-(p-Methyl)phenylprotopalmatine choride (8c)

Yield: 74%; Yellow-brown solid; mp 164–165°C; ^1^H NMR: δ 9.15 (s, 1H), 8.24 (d, *J* = 9.2 Hz, 1H), 8.15 (d, *J* = 9.2 Hz, 1H), 7.76 (s, 1H), 7.46−7.52 (m, 4H), 7.04 (s, 1H), 4.25 (t, *J* = 6.4 Hz, 2H), 4.00 (s, 3H), 3.96 (s, 3H), 3.86 (s, 3H), 3.18 (s, 3H), 3.03 (t, *J* = 6.4 Hz, 2H), 2.48 (s, 3H); ^13^C NMR: δ 172.49, 156.94, 152.89, 152.01, 149.22, 145.44, 139.80, 138.66, 133.78, 132.22, 129.51, 129.29, 128.00, 127.93, 126.38, 124.57, 122.56, 121.47, 120.26, 111.19, 109.67, 61.02, 57.43, 56.74, 56.34, 52.45, 26.32, 21.59; HRMS-ESI: *m/z* calcd 442.20128 C_28_H_28_NO_4_Cl [M − Cl]^+^, found 442.20128.

#### 8-(1-Naphthyl)protopalmatine choride (8d)

Yield: 78%; Yellow-brown solid; mp 177–178°C; ^1^H NMR: δ 9.34 (s, 1H), 8.20−8.25 (m, 3H), 8.18 (d, *J* = 8.4 Hz, 1H), 7.84 (s, 1H), 7.76−7.80 (m, 2H), 7.63−7.67 (m, 1H), 7.47−7.49 (m, 2H), 7.02 (s, 1H), 4.13−4.35 (m, 2H), 3.99 (s, 3H), 3.94 (s, 3H), 3.85 (s, 3H), 2.93−3.05 (m, 2H), 2.77 (s, 3H); ^13^C NMR: δ 172.49, 155.06, 152.70, 152.07, 149.25, 144.94, 139.44, 134.03, 133.13, 132.42, 130.87, 130.58, 129.29, 128.21, 127.47, 126.59, 126.44, 126.00, 125.06, 124.74, 123.15, 122.16, 120.41, 111.19, 109.68, 60.78, 57.35, 56.78, 56.35, 52.40, 26.59; HRMS-ESI: *m/z* calcd 478.20128 C_31_H_28_NO_4_Cl [M − Cl]^+^, found 478.20113.

#### 8-(n-Decyl)protopalmatine chloride (8e)

Yield: 75%; Brown solid; mp 172–173°C; ^1^H NMR: δ 8.90 (s, 1H), 8.20 (d, *J* = 9.2 Hz, 1H), 8.02 (d, *J* = 9.2 Hz, 1H), 7.65 (s, 1H), 7.13 (s, 1H), 4.83 (t, *J* = 6.4 Hz, 2H), 4.07 (s, 3H), 4.05 (s, 3H), 3.93 (s, 3H), 3.88 (s, 3H), 3.18 (t, *J* = 6.4 Hz, 2H), 1.79 (t, *J* = 6.4 Hz, 2H), 1.27−1.60 (m, 16H), 0.87 (t, *J* =6 .4Hz, 3H); ^13^C NMR: δ 176.25, 161.55, 152.84, 151.86, 149.18, 146.05, 138.49, 133.20, 129.31, 125.67, 125.08, 121.62, 120.35, 111.06, 109.62, 62.04, 57.50, 56.68, 56.36, 50.23, 32.77, 31.78, 29.84, 29.48, 29.46, 29.23, 29.19, 28.43, 26.84, 22.58, 14.45; HRMS-ESI: *m/z* calcd 492.31084 C_31_H_42_NO_4_Cl [M − Cl]^+^, found 492.31064.

### Biological activity assay

18 newly synthesized analogues were evaluated for their activity against the multiplication of wild-type *M. tuberculosis* strain H_37_Rv and MRD strains by the microplate alamar blue assay (MABA) at various concentrations of 128.0, 64.0, 32.0, 16.0, 8.0, 4.0, 2.0, 1.0, 0.5, 0.25, 0.125, and 0.0625 μg/ml. RIF and INH were used as positive controls. Subsequent two-fold dilutions were performed in 100 μL of 7H9 media in the 48-well microplates. Then 100 μL of bacterial suspension was added to result in a final bacterial titer of 1 × 10^6^ CFU/mL. Plates were incubated at 37°C. At optimal time, alamar blue solution was added to the entire plate. Results were recorded at 24 h post-reagent addition. Visual MIC value was defined as the lowest concentration of drug that prevented a color change.

### Cytotoxicity activity assay

African green monkey kidney (Vero) cells (6 × 10^3^ cells/well) and human lung fibroblast (MRC-5) cells (1.2 × 10^4^ cells/well) were plated into a 96-well plates and incubated 37°C in 5% CO_2_. Sixteen hours later the cell cultures were treated with various concentrations of compounds **7f** and **7g**. Cytotoxicity was evaluated with the tetrazolium 3-(4,5-dimethylthiazol-2-yl)-2,5-diphenyltetrazolium bromide (MTT) assay at 48 h. The 50% cytotoxic concentration (CC_50_) was calculated with Reed & Muench methods. Each experiment was repeated three times.

## Conclusion

In conclusion, 18 new 8-substituted BBR derivatives were synthesized and evaluated for their antimycobacterial activities against *M. tuberculosis* H_37_Rv. SAR analysis revealed that (i) introduction of a *n*-decyl at the 8-position might significantly enhance the activity; (ii) 10,11-dimethoxy on the ring D might be beneficial for the antimycobacterial activity. Among the test compounds, compound **7g** exhibited the strongest activity against both drug-susceptible strains and MDR isolates of *M. tuberculosis*, suggesting a novel mechanism of action. It has been selected as an ideal compound lead against TB for further SAR investigation. We consider 8-*n*-decylberberines to be a novel family of anti-tubercular agents with an advantage of inhibiting MDR strains of *M. tuberculosis*.

## Competing interests

The authors declare that they have no competing interests.

## Authors’ contributions

The current study is an outcome of the constructive discussion with DQS and YXW, who offered necessary guidance to YHL and HGF to carry out their synthesis and characterization experiments. FS and LMG performed the antimycobacterial activities against *M. tuberculosis* H_37_Rv tests, ST and CWB carried out the ^1^H NMR and ^13^C NMR spectral analyses and HRMS analysis, and YHL did the cytotoxicity experiment. All authors read and approved the final manuscript.

## Supplementary Material

Additional file 1 **Supporting information.** Selected copies of spectrum (1H-NMR, 13C-NMR and HRMS) for the two representative compounds. Click here for file
