# Supplementary material for: Synthesis and structure−activity relationship of 8-substituted protoberberine derivatives as a novel class of antitubercular agents
Source: Chem Cent J. 2013 Jul 10;7:117. doi: 10.1186/1752-153X-7-117 (PMC3712002; doi:10.1186/1752-153X-7-117)

^1^H NMR spectra of compound **7f**


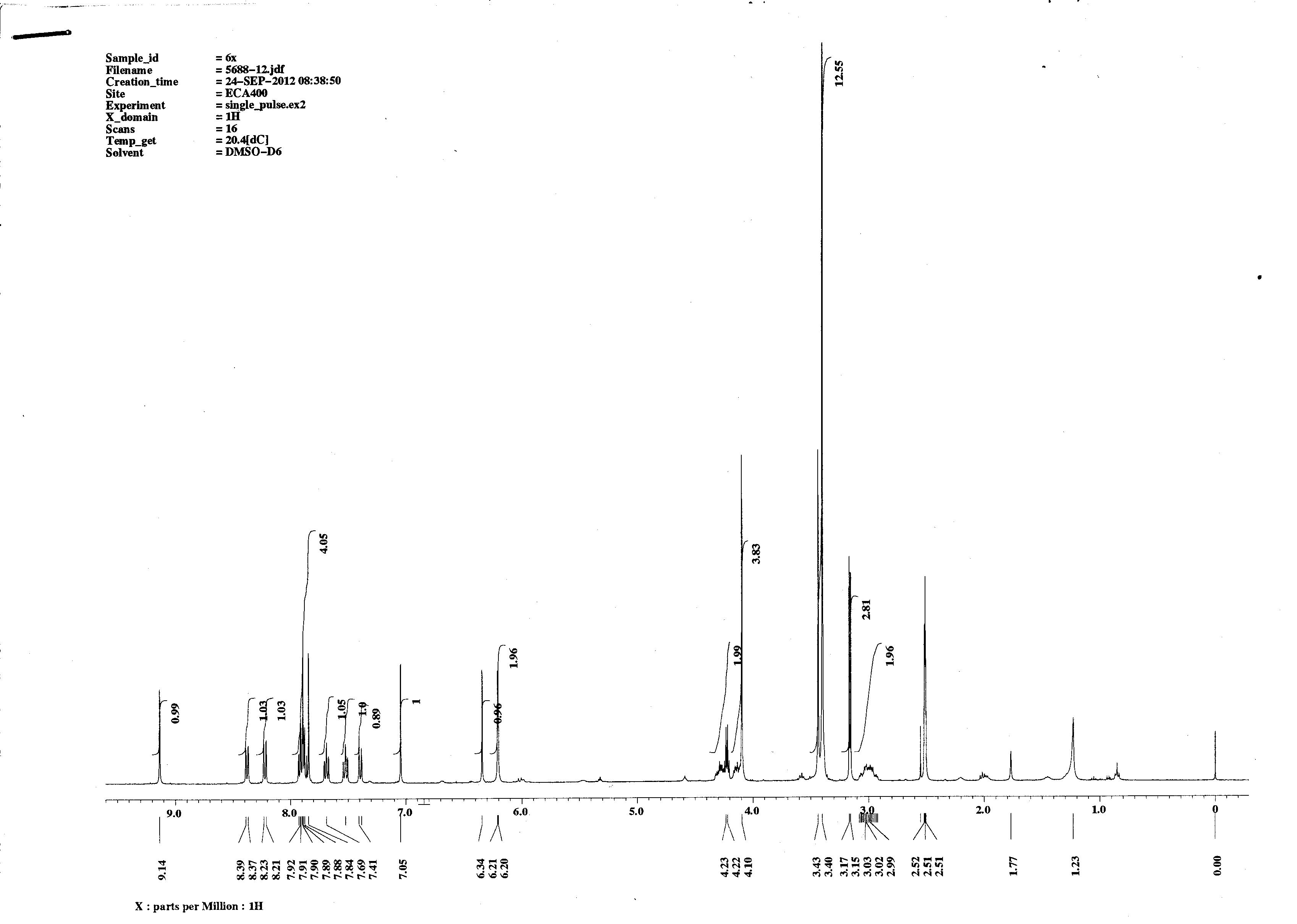


^13^C NMR spectra of compound **7f**


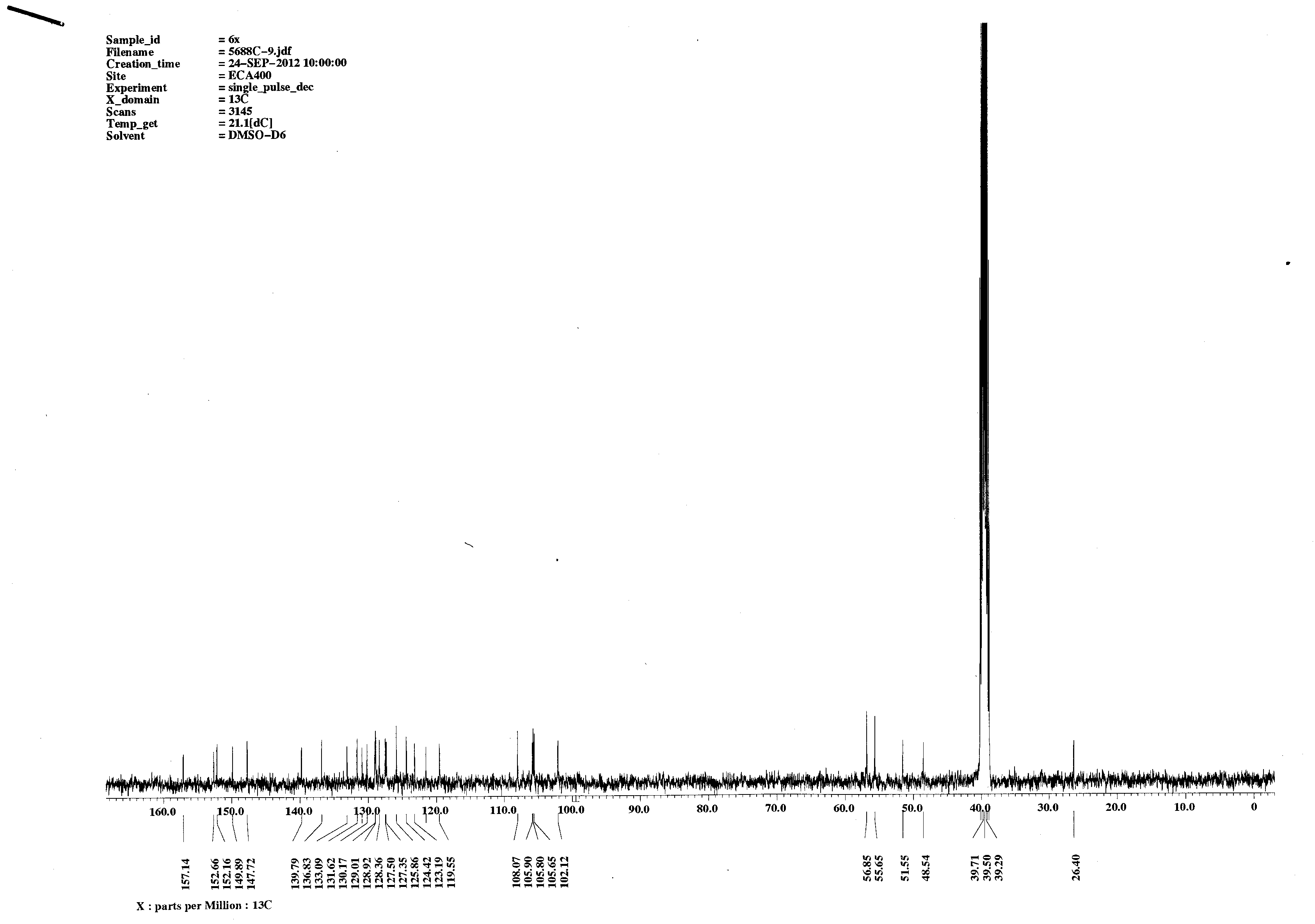


HRMS of compound **7f**


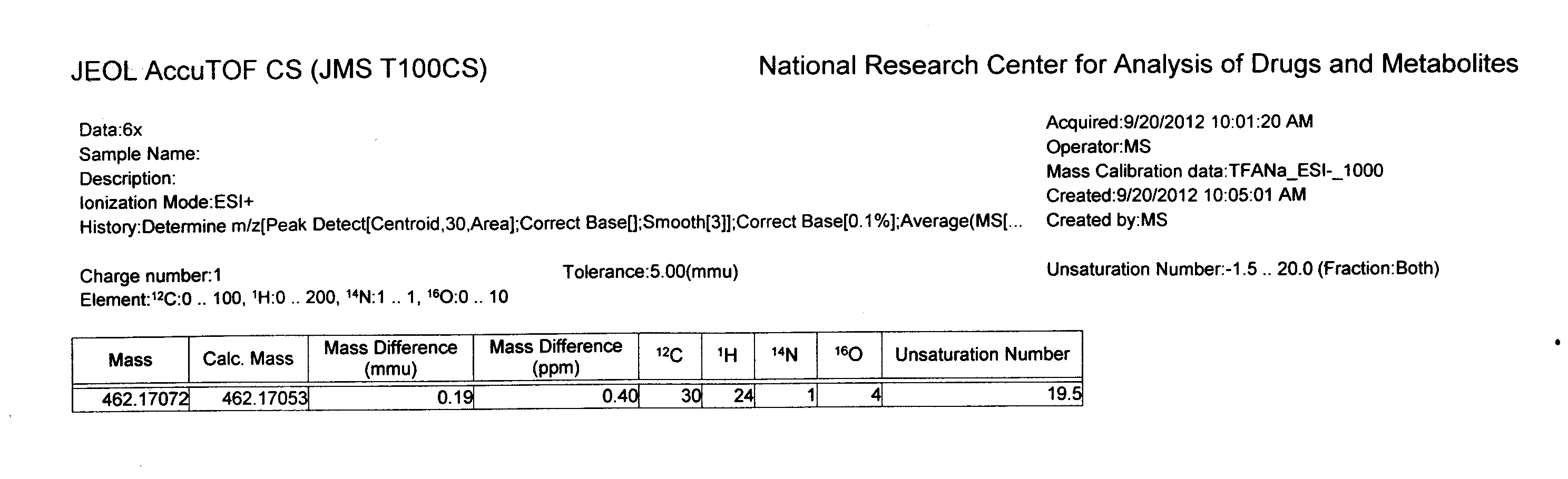


^1^H NMR spectra of compound **7g**

^13^C NMR spectra of compound **7g**

HRMS of compound **7g**


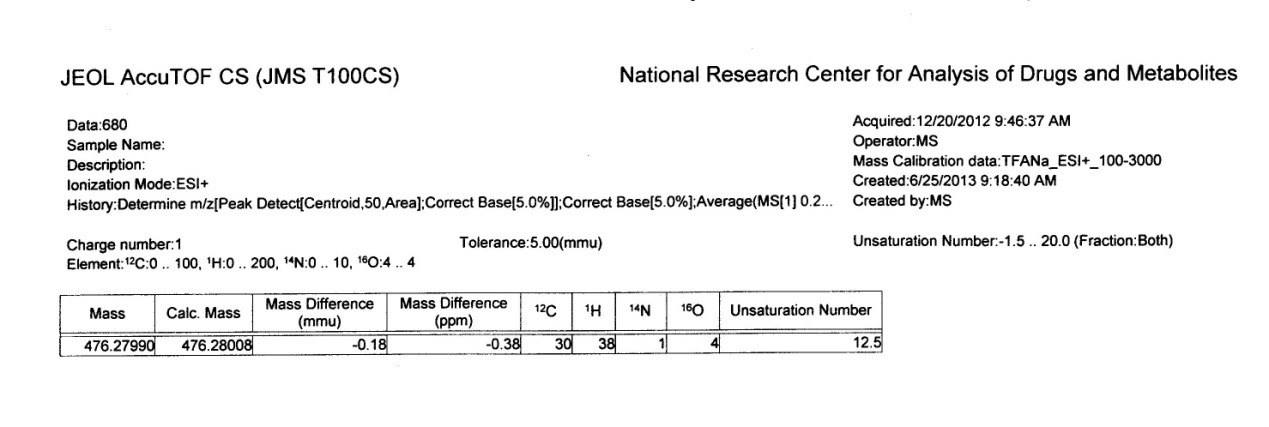

Supplement: Additional file 1 — Supporting information. Selected copies of spectrum (1H-NMR, 13C-NMR and HRMS) for the two representative compounds. [file 1752-153X-7-117-S1.docx]
